# Visual Mismatch Negativity: A Mini-Review of Non-pathological Studies With Special Populations and Stimuli

**DOI:** 10.3389/fnhum.2021.781234

**Published:** 2022-02-16

**Authors:** István Czigler, Petia Kojouharova

**Affiliations:** Institute of Cognitive Neuroscience and Psychology, Research Centre for Natural Sciences, Budapest, Hungary

**Keywords:** visual mismatch negativity (vMMN), gender, mental fatigue, hypoxia, Internet addiction, physical exercise, fine arts

## Abstract

In this mini-review, we summarized the results of 12 visual mismatch negativity (vMMN) studies that attempted to use this component as a tool for investigating differences between non-clinical samples of participants as well as the possibility of automatic discrimination in the case of specific categories of visual stimuli. These studies investigated the effects of gender, the effects of long-term differences between the groups of participants (fitness, experience in different sports, and Internet addiction), and the effects of short-term states (mental fatigue and hypoxia), as well as the vMMN effect elicited by artworks as a special stimulus category.

## Introduction

Stimuli different from the representation of expected events automatically elicit the visual mismatch negativity (vMMN)^1^ component of event-related potentials (ERPs) even if the events are unattended. VMMN emerges at posterior electrode locations within the post-stimulus range of 100–350 ms. This component is usually recorded in passive oddball paradigms in which vMMN-related stimuli are not involved in the ongoing task and is identified as the ERP difference between rare (deviant) and frequent (standard) stimuli. VMMN appears to deviant simple visual features such as color (Athanasopoulos et al., 2010) and spatial frequency (Heslenfeld, 2003), perceptual categories [e.g., symmetrical vs. random patterns (Kecskés-Kovács et al., 2013)], complex categories such as facial emotions (Astikainen and Heitanen, 2009), and lexical processing (Wei et al., 2018) (for comprehensive reviews, refer to Kimura et al., 2011; Stefanics et al., 2014). As in many fields of ERP research, attempts to apply the results from basic research concentrated on the comparison of various clinical populations (for a review, refer to Kremláček et al., 2016) and on investigating aging effects (Sulykos et al., 2017). The aim of this mini-review was to show how vMMN can be a tool for investigating differences between other, non-clinical samples of participants. Furthermore, vMMN can be a feasible tool to investigate the sensitivity to specific categories of visual stimuli, which is another promising application of ERP research. In other words, the aim was to highlight in what research areas there is emerging evidence of the usefulness of vMMN beyond the study of pathology, aging, single deviant features, sequential deviancies, and categorization *per se* (i.e., when not used as the means to study non-clinical samples), all of which have already been subject to reviews (Czigler, 2014; Stefanics et al., 2014; Kremláček et al., 2016), and lexical processing (this topic deserves a separate review). To this end, in this mini-review, we summarized research dealing with vMMN differences between different non-clinical samples, i.e., gender, expertise in particular fields, and with vMMN emerging to the uncommon type of stimuli. Accordingly, we do not review the articles on topics dealing with stimulus features and categorization (as described above), language-related effects, and effects specific to stimulation (e.g., face-specific effects, Wang et al., 2014). Studies that were published within the period of 2000–2021 (i.e., a period of considerable research activity in this field) were collected from the Web of Sciences and Scopus databases. We used the search term “visual mismatch negativity” (exact phrase search) and then chose all studies describing non-clinical samples or specific categories of visual stimuli according to the “beyond” criteria. An overall summary of the methods and results of the studies can be found in Table 1. A more detailed summary of the results including effect sizes can be found in Table 2.

**Table 1:** A summary of the review studies.

**Deviancy**	**Study**	**Deviant-standard**	**Task**	**Paradigm**	***N***	**Reference**	**VEP measurement**	**Results**
**Gender**								
Simple visual features	Langrová et al., 2012	Standard and deviant (6%) moving gratings with direction difference	Detection of target grating (6 %)	Three-stimulus oddball; stimulus duration: 200 ms, ISI: 600 ms	21 women 21 men	Right earlobe	Occipital locations	Negativity in the 120–240 ms range. No gender difference
	Yang et al., 2016	Bilateral stimuli, duration deviant (20%), 50 or 150 ms, reverse control	Detection of the changes in a fixation cross	Oddball; stimulus duration: 50 or 150 ms, ISI: 450 ms	21 women 21 men	Tip of nose	Parietal and occipital locations	Negativity for stimulus increment, 180–260 ms range. Larger in men with right side dominance
Emotion	Xu et al., 2013	Bilateral stimuli, happy, sad, and neutral faces, reverse control	Detection of the changes in a fixation cross	Oddball; stimulus duration: 150 ms, ISI: 450 ms	14 women 15 men	Tip of nose	Posterior locations (Pz, PO7, CB1, P8, PO8, CB2)	Negativity; 120–230 ms range: women: sad > happy on the right side; 230–350 ms range: sad > happy
	Li Q. et al., 2018	Cropped photographs, 4 photographs on each stimulus, happy and neutral, and fearful and neutral stimuli, deviant (20%), reverse control	Detection of the changes in a fixation cross	Oddball; stimulus duration: 200 ms, ISI: 450–600 ms	19 women 19 men	Tip of nose	P7, P8, FCz, Cz	100–200 ms and 250–300 ms: Happy-neutral sequences: women > men. Fear-neutral sequences: no gender difference
	Zhang J. et al., 2018	Bilateral stimuli, schematic sad and neutral, and happy and neutral faces, deviant (20%), reverse control	Detection of the changes in a fixation cross	Oddball; stimulus duration: 150 ms, ISI: 450 ms	16 women 18 men	Tip of nose	64 locations, extended 10–20 system. Time-frequency analysis (delta, theta, alpha, beta, gamma bands), phase lag index for connectivity measurement	150–200 ms range: women: greater alpha power to sad faces, stronger connections, wider areas involved in the response to emotional stimuli, more long connections.
Attractiveness	Zhang S. et al., 2018	Photographs of the opposite-gender faces, attractive deviant (20%) and less attractive standard models	Decision about tone frequency	Cross-modal delayed-response paradigm; stimulus duration: 100 ms, inter-tone interval: 300–770 ms, ISI: 650–700 ms, inter-trial interval: 1,500 ms	Exp. 1: 29 women, menstrual period, 31 men; Exp. 2: 30 women, ovulation period; 30 men	Tip of nose	P7, PO7, O1, P8, PO8, O2. Peak amplitudes within the 110–240, 240–380, and 380–520 ms epochs	Men: larger vMMN in the 110–240 ms range; women: larger vMMN in Exp. 2 (ovulation) than in Exp. 1 (menstruation)
**States**								
Fatigue	Li J. et al., 2016	Bilateral squares, duration deviant, 50 ms standard, 150 ms deviant (20%).	Detection of the changes in a fixation cross	Oddball; fatigue: 2.5 h of continuous performance task; ISI: 500 ms	12 women 12 men	Linked nose-lobes	Fz, Cz, Oz, O1, O2; Main amplitude and latency within the 100–300 ms range. Measurement before and after the fatigue/leisure period	Amplitude decrease in the second measurement, but larger decrease for participants in the fatigue condition
Hunger	Sultson et al., 2019	Pictures of two high fat savory or two sweet meals (deviant, 10%, and equal probability control), pictures of non-food (deviant, 10% or standard, and equal probability control). Four identical pictures on each stimulus; hunger session: refrain eating for 10–12 h and no breakfast	2-back letter matching	Oddball; stimulus duration: 450 ms, ISI: 250 ms	18 women	Earlobes	O1, Oz, O2; mean activity within the 100–160 and 160 ms ranges	Deviant-minus-equal probability control: no hunger-related difference; deviant-minus-standard: non-food deviant: no effect, food deviant: larger vMMN in “hunger” condition. Larger effect for hamburger as deviant
Hypoxia	Blacker et al., 2021	Peripheral green-black and red-black checkerboards, deviant (10%), reverse control	Visual tracking task	Oddball; hypoxia and normoxia sessions; stimulus duration: 100 ms, ISI: 500 ms	24	Right mastoid	Set of parieto-occipital/occipital and anterior locations	Hypoxia: amplitude reduction of posterior negativity (vMMN) and deviant-related anterior positivity
**Dispositions**								
Fitness	Pesonen et al., 2019	Oblique bars, deviant tilted to the left (10%) or standard tilted to the right	Auditory play	Oddball; stimulus duration: 100 ms, ISI: 1,100 ms	16 pairs of male twins; a physically more and a less active member	Cz	F3, F4, O1, Pz, O2 Integrated rectified activity within the 100–300 ms range	Physically active participants: shorter vMMN latency, and larger but not significantly activity
Sport	Petro et al., 2021	Bilaterally presented checkerboards, reversed location of deviants (10%) and standard checkerboards, reverse control	Visual tracking	Oddball; stimulus duration: 100 ms, ISI: 530 +/-50 ms	20 target shooters, 20 handball players	Tip of nose	Fz, P7, P8, PO3, PO4, PO7, PO8, POz, O1, Oz, O2; main amplitude within the 100–150 ms range	Larger vMMN in the handball players over the posterior locations
Addiction^*^	He et al., 2018	Bilaterally presented network logos or control pictures in separate sequences; deviants: (20%) red pictures	Detection of the changes in a fixation cross	Oddball; duration: 100 ms, ISI: 500 ms	15 internet addicts, 15 controls	Tip of nose	O1, Oz, O2, PO5, PO6; average of 200–300 ms range	Internet-related deviant logos elicited larger vMMN in the addict group than the control pictures
**Stimuli**								
Aesthetics	Menzel et al., 2018	20 non-figural artworks and their shuffled versions; reverse control procedure, 20% deviants	Detection of the changes in a fixation cross	Oddball; stimulus duration: 400 ms, ISI: 500–700 ms	17	Offline re-referenced to average	40 ms deviant-minus-control difference, at least two neighboring locations	In the 220–300 ms range deviant originals elicited positive, shuffled deviants elicited negative potential differences

* *Addictive internet usage*.

**Table 2:** A summary of the effect sizes for intergroup comparisons in the review studies.

**Study**	**Comparison**	**Measure**	**Channel/ region/band**	**Interval**	**Statistics**	**Reported effect size**	***SMD* (*Cohen's d* [95% CI])**	***MD***
Langrová et al. (2012)	Difference in vMMN between the two groups	Area under the curve	Occipital	120–240 ms	Wilcoxon signed-rank test^*^	NA	NA	39.1 μV (more negative in the female group, median difference!)
Yang et al. (2016)	vMMN in the stimulus increment condition between groups with hemisphere and site as within-subject factors^1^	Mean amplitude	P3/O1, Pz/Oz, P4/O2	180–260 ms	*F* (gender)	NA	1.0399 [0.6089, 1.4708]^a^	0.71 μV (more negative in the male group)
					*F* (gender × hemisphere)	NA	Over the midline 0.6354 [0.0155, 1.2553]^b^ Over the right hemisphere 0.809 [0.1799, 1.4381]^b^	NA (more negative in the male group)
		Peak latency			*F* (gender)	NA	NA	17 ms (longer in the female group)
Xu et al. (2013)	vMMN to happy vs. vMMN to sad faces between groups with hemisphere and site as further within-subject factors	Mean amplitude	P7, P8, PO7, PO8, CB1, CB2	120–230 ms	*F* (gender)^*^	$\eta_{\text{p}}^{2}$ = 0.041	0.4139 [−0.3222, 1.15]^a^	0.35 μV (more negative in the male group)
					*F* (gender × stimulus type × hemisphere)	$\eta_{\text{p}}^{2}$ = 0.141	Over the right hemisphere: sad faces: −0.1546 [−0.8841, 0.5748]^a^^+^ happy faces: 0.6469 [−0.1002, 1.3941]^a^^+^ Over the left hemisphere: sad faces: 0.099 [−0.6298, 0.8278]^a^^+^ happy faces: 0.3119 [−0.4208, 1.0447]^a^^+^	Over the right hemisphere: sad faces: 0.21 μV (more negative in the female group)^+^ happy faces: 0.95 μv (more negative in the male group)^+^ Over the left hemisphere: sad faces: 0.14 μV (more negative in the male group)^+^ happy faces: 0.51 μV (more negative in the male group)^+^
				270–350 ms	*F* (gender)^*^	$\eta_{\text{p}}^{2}$ = 0.093	0.6514 [−0.096, 1.3988]^a^	0.61 μV (more negative in the male group)
Li Q. et al. (2018)	vMMN to happy faces vs. vMMN to neutral faces between groups with hemisphere as a further within-subject factor	Mean amplitude	Occipitotemporal (P7, P8)	100–200 ms	*F* (gender)	$\eta_{\text{p}}^{2}$ = 0.11	NA	NA
				250–350 ms	*F* (gender)	$\eta_{\text{p}}^{2}$ = 0.17	NA	NA
					*F* (gender × emotion × hemisphere)	$\eta_{\text{p}}^{2}$ = 0.11	NA	NA
	vMMN to happy faces vs. vMMN to neutral faces between groups with site as a further within-subject factor		Fronto-central (FCz, Cz)	100–200 ms 250–350 ms	*F* (gender) F (gender)	$\eta_{\text{p}}^{2}$ = 0.1 $\eta_{\text{p}}^{2}$ = 0.14	NA NA	NA NA
	vMMN to fearful faces vs. vMMN to neutral faces between groups with hemisphere as a further within-subject factor		Occipitotemporal (P7, P8)	100–200 ms 250–350 ms	*F* (gender)^*^ *F* (gender)^*^	NA NA	NA NA	NA NA
	vMMN to fearful faces vs. vMMN to neutral faces between groups with site as a further within-subject factor		Fronto-central (FCz, Cz)	100–200 ms 250–350 ms	*F* (gender)^*^ *F* (gender)^*^	NA NA	NA NA	NA NA
Zhang J. et al. (2018)	Deviant vs. standard comparison (vMMN) for happy faces between groups with hemisphere and site as further within-subject factors	Mean amplitude	Occipitotemporal (P7, P8, PO7, PO8, CB1, CB2)	130–210 ms	*F* (gender)^*^	NA	NA	NA
	Deviant vs. standard comparison (vMMN) for sad faces between groups with hemisphere and site as further within-subject factors				*F* (gender)^*^	NA	NA	NA
	Phase-locked neural activity between groups with emotion as a further within-subject factor	TF power	Alpha band	150–250 ms	*F* (gender × emotion)	NA	NA	NA
	Functional connectivity for happy faces between groups^2^	PLI (phase lag index)			*F* (gender)	NA	0.8179 [0.117, 1.5189]^b^	NA
	Functional connectivity for sad faces between groups^2^				*F* (gender)	NA	0.885 [0.1795, 1.5905]^b^	NA
	Brain network connectivity for happy faces between groups^3^	Average degree			Independent *t*-test	NA	0.7522 [0.0554, 1.4489]^c^	NA
	Brain network connectivity for sad faces between groups^3^				Independent *t*-test	NA	0.9275 [0.2189, 1.6361]^c^	NA
	Length of the connections for happy faces between groups^3^	DCT (Difference in the change trends)			Independent *t*-test	NA	0.7099 [0.0156, 1.4041]^c^	NA
	Length of the connections for sad faces between groups^3^				Independent *t*-test	NA	0.7065 [0.0124, 1.4005]^c^	NA
Zhang S. et al. (2018)	vMMN to attractive faces between groups (men and FM) with hemisphere and site as further within-subject factors (Experiment 1)	Mean amplitude	O1, O2, PO7, PO8, P7, P8	100–240 ms	*F* (gender)	$\eta_{\text{p}}^{2}$ = 0.809	NA	3.53 μV (more negative in the male group)
				240–380 ms	*F* (gender)	$\eta_{\text{p}}^{2}$ = 0.648	NA	1.584 μV (more negative in the male group)
				380–520 ms	*F* (gender)^*^	NA	NA	0.194 μV (more negative in the male group)
		Peak amplitude		100–520 ms	Independent *t*-test	NA	3.9736 [3.1007, 4.8464]^c^	3.53 μV (more negative in the male group)
		Peak latency		100–520 ms	Independent *t*-test	NA	2.5736 [1.8892, 3.258]^c^	58 ms (longer in the female group)
	vMMN to attractive faces between groups (men and FM) with hemisphere and site as further within-subject factors (Experiment 2)	Mean amplitude	O1, O2, PO7, PO8, P7, P8	100-240 ms	*F* (gender)	$\eta_{\text{p}}^{2}$ = 0.555	NA	2.603 μV (more negative in the male group)
				240–380 ms	*F* (gender)^*^	NA	NA	0.074 μV (more negative in the male group)
				380–520 ms	*F* (gender)	$\eta_{\text{p}}^{2}$ = 0.907	NA	4.223 μV (more negative in the female group)
		Peak amplitude		100–520 ms	Independent *t*-test	NA	0.2863 [−0.2223, 0.795]^c^	0.296 μV (more negative in the male group)
		Peak latency		100–520 ms	Independent *t*-test	NA	9.7909 [7.9675, 11.6143]^c^	238 ms (longer in the female group)
	vMMN to attractive faces between groups with hemisphere and site as further within-subject factors (Experiment 1 and Experiment 2 pooled)^4^	Mean amplitude	O1, O2, PO7, PO8, P7, P8	100–240 ms	*F* (gender)	$\eta_{\text{p}}^{2}$ = 0.699	NA	0.927 μV (more negative in the FO compared to the FM group)
				240–380 ms	*F* (gender)	$\eta_{\text{p}}^{2}$ = 0.574	NA	1.313 μV (more negative in the FO compared to the FM group)
				380–520 ms	*F* (gender)	$\eta_{\text{p}}^{2}$ = 0.922	NA	4.417 μV (more negative in the FO compared to the FM group)
		Peak amplitude		100–520 ms	*F* (gender)	$\eta_{\text{p}}^{2}$ = 0.77	NA	3.227 μV (more negative in the FO compared to the FM group)
		Peak latency		100–520 ms	*F* (gender)	$\eta_{\text{p}}^{2}$ = 0.944	NA	180 ms (longer in the FO compared to the FM group)
Li J. et al. (2016)	vMMN between groups pre- and post-manipulation	Mean amplitude	Fz, Cz, O1, O2, Oz	100–360	NA	NA	NA	pre-manipulation only: 0.19 μV (more negative in the “fatigue” group)
	vMMN between groups pre- and post-manipulation with site as a further within-subject factor	Maximum amplitude		100–300 ms	*F* (group)	$\eta_{\text{p}}^{2}$ = 0.71	NA	NA
					*F* (group × time)	$\eta_{\text{p}}^{2}$ = 0.83	NA	Post-manipulation differences between the two groups (more negative in the “leisure” group): O1: 1.811 μV O2: 2.53 μV Oz: 2.339 μV Cz: 2.252 μV Fz: 2.623 μV
		Peak latency		100–300 ms	*F* (group × time)	$\eta_{\text{p}}^{2}$ = 0.19	NA	NA
Pesonen et al. (2019)	vMMN between groups	Peak latency	O1, O2, Pz	Early component (not specified)	Mann-Whitney test^*^	NA	Channel 71 (O1):−0.3712 [−1.0701, 0.3277]^a^ channel 72 (Pz): 0.0481 [−0.645, 0.7411]^a^ channel 76 (O2): = 0.0951 [−0.5982, 0.7885]^a^	Channel 71 (O1): 8 ms channel 72 (Pz): 1 ms channel 76 (O2): 2 ms (longer in the inactive group at O1, in the active at Pz and O2)
				Late component (207–248 ms)	Mann–Whitney test	NA	Channel 71 (O1):−1.3799 [−2.151, −0.6089]^a^ channel 72 (Pz): = −0.8847 [−1.6107, −0.1586]^a^ channel 76 (O2): = −0.9618 [−1.6938, −0.2299]^a^	Channel 71 (O1): 41 ms channel 72 (Pz): 28 ms channel 76 (O2): 30 ms (longer in the inactive group)
		AUC (vMMN integral)		100–300 ms	Mann–Whitney test^*^	NA	Channel 71 (O1): 0.7399 [0.0237, 1.4562]^a^ channel 72 (Pz): = 0.5759 [−0.1313, 1.283]^a^ channel 76 (O2): = 0.4981 [−0.2055, 1.2017]^a^	Channel 71 (O1): 0.11 μV channel 72 (Pz): 0.08 μV channel 76 (O2): 0.09 μV (more negative in the active group)
Petro et al. (2021)	vMMN between groups with anteriority and laterality as further within-subject factors	Mean amplitude	PO3, POZ, PO4, O1, OZ, and O2	100–150 ms	*F* (group)	$\eta_{\text{p}}^{2}$ = 0.12	NA	1.1 μV (more negative in handball players group)
He et al. (2018)	vMMN to internet-related and neutral pictures between groups with picture color and hemisphere as further within-subject factors	Mean amplitude	Oz	200–300 ms	*F* (picture × group)	η^2^ = 0.37	NA	NA
	vMMN to internet-related pictures between groups				*F* (group)	η^2^ = 0.21	−1.0107 [−1.7707, −0.2507]^a^	0.82 μV (more negative in the IA group)
	vMMN to neutral pictures between groups				*F* (group)	η^2^ = 0.15	0.818 [0.073, 1.563]^a^	0.62 μV (more negative in the control group)
	vMMN to internet-related and neutral pictures between groups with picture color and hemisphere as further within-subject factors		Occipitotemporal (O1, O2, PO5, PO6)	200–300 ms	*F* (picture × group)	η^2^ = 0.3	NA	NA
	vMMN to internet-related pictures between groups				*F* (group)	η^2^ = 0.15	−0.7913 [−1.5345, −0.0482]^a^	0.75 μV (more negative in the IA group)
	vMMN to neutral pictures between groups				*F* (group)	η^2^ = 0.16	−0.8581 [−1.606, −0.1102]^a^	0.86 μV (more negative in the control group)
Blacker et al. (2021)	vMMN in normoxia and hypoxia conditions (within-subject study)	Peak amplitude	Posterior (PO8, PO4, POz, PO7, O1, O2, O)	150–250 ms	Paired-samples *t*-test (condition)	NA	NA	Oz: 0.087 μV (more negative in normoxia);
			Frontal (Fz, FCz, Cz)		Paired-samples *t*-test (condition)	NA	NA	Fz: 0.098 μV (more positive in normoxia)
		Peak latency	Posterior (PO8, PO4, POz, PO7, O1, O2, Oz)		Paired-samples *t*-test (condition)^*^	NA	NA	Oz: 2.67 ms (longer in normoxia)
			Frontal (Fz, FCz, Cz)		Paired-samples *t*-test (condition)^*^	NA	NA	Fz: 1.88 ms (longer in hypoxia)
Sultson et al. (2019)	This was a within-subject study. There are no reported effect sizes. The reported data does not allow for calculating *MD*s.							
Menzel et al. (2018)	This was a within-subject study. There are no reported effect sizes. The reported data does not allow for calculating *MD*s.							

*Only main effects and interactions for the group factor (between-subject factor) are included (interactions were included only when statistically significant). SMD indicates standardized mean difference and is measured here with Cohen's d. For all review studies, SMD was calculated from the available data (as reported in the text or in tables). MD (mean difference) indicates the difference between the means of the groups (as reported in the text or in tables). NA indicates “not reported” or “cannot be calculated based on the available data.” The table also includes within-subject studies, for which only MDs were reported where available (in this case, MD means the difference between the means of the compared conditions)*.

a *Calculated from mean and SD or mean and SE*.

b *Calculated from the F-test value for a one-way ANOVA for two groups*.

c *Calculated from the independent t-test value or p-value*.

* *The result of the statistical test was not significant*.

+ *Only the differences within the female and the male groups are investigated further for this interaction and not the intergroup differences, and there is no information whether the between-subject differences (the SMDs and MDs calculated here) are significant*.

1 *Based on the reported degrees of freedom, e.g., F(1,42), it is possible that the calculations were conducted on 43 instead of the reported 42 participants*.

2 *Assuming that the reported F-test value is for a one-way ANOVA with two groups*.

3 *The reported p-values of the t-tests are most likely the Bonferroni corrected values*.

4 *It is not clear that the male group from which experiment was used in this comparison, but based on the degrees of freedom, it was most likely the male group from Experiment 2*.

## Gender-Related Differences in vMMN

The majority of vMMN studies include both female and male participants. Supposedly, in these studies, researchers would have detected any substantial gender-related differences, but no such incidental observations have been published. Several studies have targeted directly possible gender-related differences in vMMN to simple visual features, facial emotions, and attractiveness.

In the first direct study (Langrová et al., 2012), age-matched young women and men participated in a three-stimulus oddball design (within a sequence, there were frequent and infrequent non-target and infrequent target stimuli). The target stimuli were centrally presented sinusoidal grating patterns, whereas vMMN-related moving sinusoidal gratings were presented at the periphery. The upward movement direction was standard, and the downward movement direction was deviant. vMMN emerged within the latency range of 120–240 ms without any gender-related differences.

Yang et al. (2016) investigated the duration-related vMMN in 21 female and 21 male participants. They applied the reverse control procedure where both values of the stimuli were standard and deviant; therefore, stimuli with the same physical parameters were compared. Stimulus durations were 50 and 150 ms, respectively. Stimuli were black squares presented on the two sides of a fixation cross. Participants had to attend and react to the occasional size change of the cross. VMMN was analyzed at the P3, O1, Pz, Oz, P4, and O2 locations. The gender-related vMMN differences appeared for the mean amplitude in the range of 180–260 ms. In this study, vMMN was larger overall as well as asymmetric in male participants, with the mean amplitude being larger over the right hemisphere.

Many studies have indicated gender-related differences to emotional stimuli, both at behavioral and electrophysiological levels, according to which women seem to be more sensitive to various aspects of emotions (for reviews, refer to Vuilleumier and Pourtois, 2007; Whittle et al., 2011). A reasonable question is whether these differences can be observed at a preattentive level. So far, the effect of facial emotions has been investigated in four vMMN studies.

Xu et al. (2013) presented neutral, happy, and sad schematic faces to young female (*n* = 14) and male (*n* = 15) participants in a reverse control procedure (only happy and neutral or sad and neutral faces were presented together). A face was presented on each side of a fixation cross. The task was to react to the changes of the cross. VMMN was calculated as the average activity of the potential differences in the ranges of 120–230 and 230–350 ms over parietal and occipitoparietal locations. Only vMMN to happy and sad faces were compared. In the range of 120–230 ms, the vMMN amplitude was larger over the right hemisphere to sad than to happy faces in the female group, while there were no emotion-related differences in the male group. In the range of 230–350 ms, sad faces elicited a larger vMMN than happy faces in both gender groups.

In a more recent study, Li Q. et al. (2018) presented photographs of happy, fearful, and neutral faces to young female and male participants (*n* = 19 in both groups) in a reverse control procedure (only happy and neutral or fearful and neutral faces were presented together). Four cropped photographs appeared in the four corners of an imaginary square, and participants had to detect changes in a fixation cross at the center of the screen. VMMN was analyzed at P7, P8, FCz, and Cz locations as the mean activity in the ranges of 100–200 and 250–350 ms and was compared separately for happy vs. neutral and fearful vs. neutral faces. In the happy-neutral context, a larger vMMN to both happy and neutral faces was elicited in the female group compared to the male group in both latency ranges. In the later latency range, this difference appeared in both hemispheres for happy faces but only in the right hemisphere for neutral faces. There were no gender-related differences in the fearful-neutral context in either of the latency ranges.

Zhang J. et al. (2018) investigated functional connectivity to deviant-minus-standard ERP differences using schematic sad, happy, and neutral faces (only happy and neutral or sad and neutral faces were presented together). The participants were 16 young women and 18 young men. Deviant and standard stimuli were delivered in a reverse control arrangement. Two identical faces appeared on the two sides of a fixation cross. The participants had to detect occasional size changes of the cross. To obtain data on functional connectivity, the time-frequency analysis (delta, theta, alpha, beta, and gamma bands) was followed by the calculation of the phase lag index. The latter was used to calculate short and long connections. Only the happy and sad face conditions were compared. The gender-related differences in emotional processing appeared in the range of 150–250 ms, with alpha activity to sad stimuli being greater compared to happy faces in the female group. The connections were stronger to both emotional stimuli in women than in men. The connections in men were confined to occipital areas and to a frontal area, while in women, the network involved wider occipital, parietal, and frontal sites. In women, the number of long connections was larger than in men, both within and between hemispheres.

Another evolutionarily important aspect of the face is *attractiveness*. Zhang S. et al. (2018) investigated vMMN to attractive opposite-gender faces in women and men (i.e., photographs were preselected as attractive or less attractive portraits). In Experiment 1 (29 women, 31 men), the female participants were investigated during the menstrual phase, while in Experiment 2 (30 women, 30 men), the female participants were in the ovulation period. The authors hypothesized that the effects of male attractiveness increased during ovulation. Instead of the traditional oddball design, the cross-modal delayed-matching procedure was applied. The participants had to discriminate the pitch of sounds and respond after the onset of an imperative click. Between the two sounds, 0–2 task-irrelevant photographs were presented. Less attractive photographs were frequent (standard), and attractive ones were rare (deviant). The amplitudes of the deviant-minus-standard potential differences were measured in the ranges of 100–240, 240–380, and 380–520 ms. VMMN was measured as the largest negativity at posterior locations. In the earlier and middle ranges, vMMN was larger in the male participants in both experiments. In Experiment 2, in the late range (380–520 ms), the difference was larger in the female group. Comparing the female participants in the two studies, in the early and late ranges, vMMN was larger for participants in the ovulation phase. Accordingly, attractiveness has a generally larger effect in men, but in periods of fertility, the role of attractiveness was increased in female participants. The late range was longer than the usual range of vMMN. Due to the not particularly strict attention control in the cross-modal delayed-response paradigm, it is doubtful that the ERPs in the late range are the signatures of automatic change detection.

## Transient States

The three review studies dealt with mental fatigue, hunger, and hypoxia, respectively. Li J. et al. (2016) investigated young participants divided into “fatigue” or “leisure” groups (*n* = 12 in both groups). Mental fatigue was elicited by performing a continuous detection task for 2.5 h. The control (“leisure”) group was free to engage in self-chosen activities. Mental fatigue and mood were assessed by questionnaires pre- and post-manipulation, and these results indicated differences between the groups. VMMN was assessed in a duration oddball task with 50 ms (standard) and 150 ms (deviant) exposure of two black squares on the two sides of a fixation point. The task was to detect occasional size changes of the fixation point. VMMN was measured as the maximum amplitude in the range of 100–300 ms at the Fz, Cz, O1, Oz, and O2 locations. The decrease in vMMN amplitude was larger in the “fatigue” group compared to the pre-manipulation measurement. The fatigue-related decrement over the five electrodes was fairly large, i.e., 1.8 at post- vs. 4.1 μV at pre-manipulation. Peak latency in the “fatigue” group also increased between the two measures.

Sultson et al. (2019) investigated the effect of hunger on vMMN. The stimuli were the pictures of food (high-fat savory or high-fat sweet) and the pictures of non-food items (each food stimuli had a non-food counterpart). Participants (18 women) were tested in both “hunger” and “fed” conditions. Experiments were conducted in the morning. The participants were asked to refrain from eating for 10–12 h before the session. In the “fed” session, food was provided in the laboratory before the electroencephalography (EEG) recording. Within the oddball sequences, the standard was a non-food picture, and two high-fat savory or high-fat sweet (depending on the food condition) and one different non-food pictures were deviants. Equal probability control sequences (25% of each of the four stimulus types) were also administered. Four identical pictures were presented in the four corners of an imaginary square, and the task-related stimuli, i.e., items of a 2-back letter-matching task, were presented at the center. VMMN was measured at the O1, Oz, and O2 locations as the mean activity of the deviant-minus-standard and deviant-minus-control potential differences in the ranges of 100–160 and 160–220 ms. The deviant-minus-control differences did not result in a reliable vMMN. No differences between the effects of the two kinds of food deviants were found in the deviant-minus-standard differences; however, food stimuli elicited larger vMMN in the “hunger” condition. Interestingly, one of the high-fat savory foods, i.e., the hamburger, elicited a larger effect than the rest of the food stimuli.

Blacker et al. (2021) analyzed 24 young participants under normoxia (20.4% O_2_) and hypoxia (10.6% O_2_, corresponding to ~5,300 m altitude). Participants performed a tracking task with centrally presented events, while the vMMN-related stimuli, i.e., isoluminant green-black and red-black checkerboards, appeared on two lateral displays. A reverse control procedure was applied. VMMN was measured at a set of posterior (parieto-occipital and occipital) and anterior (Fz, FCz, and Cz) locations as the peak of the potential difference^2^ in the range of 150–250 ms. Amplitudes were smaller (negativity at posterior and positivity at anterior locations) in hypoxia compared to normoxia. It should be noted that vMMN amplitudes, and accordingly the differences, were small, i.e., ~0.1 μV.

## Enduring Dispositions

In this “Enduring dispositions” section, we present the data about the effect of physical fitness, expertise in sports with focal vs. distributed attentional demands, and Internet addiction on vMMN. Pesonen et al. (2019) investigated 16 pairs of male twins. One member of each pair was physically more active than the other (physically active for more vs. fewer than two times per week). The participants listened to an auditory play while oblique bars were presented as vMMN-related stimuli. One orientation of the bar was the standard, the other was the deviant. ERPs were analyzed at locations near F3, F4, O1, Pz, and O2. Over the posterior locations, the peak latency of the potential difference was shorter in the physically active group. Total vMMN activation was measured as waveform integrals in the rectified potential differences in the range of 100–300 ms. There was a tendency for larger activation in the physically active participants.

Petro et al. (2021) compared vMMN in groups of target shooters and handball players (*n* = 20 in both groups). While shooting demands well-established focal attention, handball players have to divide attention among events within a wide visual field. Checkerboard stimuli were presented bilaterally. The locations of the black and white squares were swapped as standard and deviant in a reverse control design. The participants performed a tracking task to move stimuli within a central field. ERPs were measured at the FZ, P7, P8, PO3, PO4, PO7, PO8, POZ, O1, O2, and OZ locations. VMMN was calculated as the mean amplitude in the range of 100–150 ms. Over the posterior electrodes, vMMN was larger in the handball player group, i.e., athletes with demand for processing events from a wide visual field were more sensitive to peripheral events violating sequential regulations. The difference was fairly large over the occipital locations, i.e., −2.3 vs. −1.2 μV.

He et al. (2018) investigated 15 Internet addicts and 15 control participants [i.e., Internet addiction is now included in the *Diagnostic and Statistical Manual of Mental Disorders, Fifth Edition* (DSM-V), but it was not discussed in the review by Kremláček et al. (2016)]. In this study, the stimuli were common network logos and pictures of everyday objects. These stimuli were presented on the two sides of a fixation cross. The task was to react to the occasional changes of the cross. ERPs were measured at the O1, Oz, O2, PO5, and PO6 locations, as the average of the range of 200–300 ms. Stimuli had either black or red outlines, with the red ones being the deviants. The Internet-related and the red deviants elicited larger vMMN in both groups, but the difference was reliable only in the group of Internet addicts.

## Specific Stimuli

Menzel et al. (2018) investigated whether abstract artworks are automatically distinguished from the reorganized versions of the same pictures. To this end, elements from 20 artworks were shuffled. This way, the elements were identical, but the composition became different. The pictures were presented in the lower half of the visual field. In the center, a fixation cross was presented, and the task was to react to occasional changes. Seventeen young participants were investigated in the study. After the EEG session, the participants rated the pictures on the dimensions of harmony, orderliness, and likability. VMMN was identified when the amplitude of the potential differences was different from zero at least for 20 subsequent points (40 ms) and at least over two subsequent electrode locations. Deviant-related differences appeared to both the original and the shuffled versions, but the deviant originals elicited positive differences, while the shuffled versions elicited negative differences. Such differences appeared in various ranges between 146 and 871 ms, but independent of the polarity, they concentrated in the range of 220–300 ms at parietal and parieto-occipital locations. While the two picture versions were similar, the participants rated the originals as more harmonious and ordered.

## Discussion

We aimed to review studies using vMMN in non-clinical populations, the application of vMMN under specific circumstances, and investigate the processing of specific visual stimuli. This way, we attempted to demonstrate the wide range of utilizations of this ERP component. VMMN seems to be sensitive enough to disclose gender differences as well as both the long-term and temporary differences between the non-clinical samples of participants. However, due to the publication bias, it is unknown that how many studies without reliable differences have remained unpublished.

As the results of the reviewed studies show, women are more sensitive to emotional events even if these events are outside the focus of attention. This is in agreement with the results of studies with various methods (e.g., Vuilleumier and Pourtois, 2007; Whittle et al., 2011). It is an open question whether the gender difference is confined to the negative (sad; Xu et al., 2013) or positive (happy; Li Q. et al., 2018) emotions. This larger sensitivity is indicated by the involvement of wider brain activity and a larger number of long connections (Zhang J. et al., 2018).

To compare the role of the attractiveness of opposite-gender faces, according to the investigated sample of stimuli and sample of participants, Zhang S. et al. (2018) obtained larger sensitivity in men. However, sensitivity to attractiveness in the female participants increased during the fertility period.

A considerable vMMN amplitude reduction appeared in the case of mental fatigue (Li J. et al., 2016). Accordingly, vMMN seems to be a promising indicator in this field. Hunger increased sensitivity to food-related deviant stimuli (Sultson et al., 2019), but instead of nutrition quality, the saliency of particular foods (hamburger in the study) elicited the largest effect. In the case of hypoxia, vMMN amplitude decreased (Blacker et al., 2021), but the difference was small. Regular physical activity had no effect on vMMN amplitude (Pesonen et al., 2019), but it reduced vMMN latency. Athletes from sports that demand the processing of a wider visual field were more sensitive to changes in the periphery than athletes from sports with strong focal attention demand (Petro et al., 2021). It is unknown whether this is an effect of practice or a part of trait differences. As Menzel et al. (2018) pointed out, vMMN can be a useful tool even in experimental aesthetics.

On a methodological level, the majority of studies presented the vMMN-related stimuli in two or four locations around the center of the visual field. In the most popular task, the participants had to detect changes in the fixation point. In principle, such changes can be introduced at any moment within the sequence. However, in the previous studies, the task-related events appeared only during the interstimulus interval. As we argued earlier (Czigler, 2007), in such an arrangement, participants can discover that, during the presentation of vMMN-related stimuli, there are no task-related changes; therefore, from time to time, they can observe the task-irrelevant events. Only a few studies applied continuous tasks such as tracking (Blacker et al., 2021; Petro et al., 2021) or a 2-back task (Sultson et al., 2019). What is really needed is research comparing vMMN to identical deviants in sequences with various task-related events.

Finally, what is the desirable progress in the field? In all studies, the differences appeared between various samples or between various conditions. The next step is to develop methods appropriate for use in applied research. Fatigue, hypoxia, and sensitivity to peripheral stimuli are the fields that require the methods of objective assessment of individual differences, and it seems that vMMN is a promising tool for fulfilling this purpose.

## Author Contributions

IC and PK drafted and wrote the manuscript. All authors contributed to the article and approved the submitted version.

## Funding

This study was financially supported by OTKA-K funding scheme (No. 119587) provided by the Ministry of Innovation and Technology of Hungary through the National Research, Development, and Innovation Office.

## Conflict of Interest

The authors declare that the research was conducted in the absence of any commercial or financial relationships that could be construed as a potential conflict of interest.

## Publisher's Note

All claims expressed in this article are solely those of the authors and do not necessarily represent those of their affiliated organizations, or those of the publisher, the editors and the reviewers. Any product that may be evaluated in this article, or claim that may be made by its manufacturer, is not guaranteed or endorsed by the publisher.

## Footnotes

^1^VMMN is the term found in the relevant literature. However, in several studies, the component has positive polarity (Amado and Kovács, 2016).

^2^The paper states the difference as standard-minus-deviant, but the authors corrected the text on p. 329, and they subtracted the ERPs to the standard from the ERPs to the deviant.
